# “So-called” Spinal Menigitis

**Published:** 1892-08

**Authors:** A. W. Clement

**Affiliations:** Baltimore


### "SO-CALLED" SPINAL MENINGITIS.

By A. W. Clement, D. V. S.

Through the kindness of Dr. Dougherty I was enabled to make several autopsies on horses which had died in a car-stable here, where the disease in question affected quite a number of animals.

The clinical history of these cases as given by Dr. Dougherty is, in general, as follows: The animals were all attacked suddenly, some while standing in the stall feeding, others while at work. They would begin to totter and in a very few minutes would be down and unable to rise. There was complete sudden loss of power over the hind extremities, followed later by partial loss of power of the fore extremities, and finally unable in some cases to raise their head for some time before death. The animals seemed to be quite conscious throughout and would eat heartily of hay even after their head had to be supported to do so. The eyes were bright and the pupils dilated. The visible mucous membranes were hyperæmic. Pulse rather slow and wiry. Temperature in all cases a little below normal. The urine was passed involuntarily and was dark in color. The penis was hanging out of the sheath and very much swollen.

Twelve horses were affected in a stable containing 450. Those affected were in different parts of the stable. All of the horses were fed and cared for alike. Of the number attacked (twelve), but one fully recoved, and he only after several weeks. Death occurred in from one to three days after the beginning of the attack The one which recovered was a milder case and was kept in sling from the beginning of the attack.

Autopsy, Case No. 1.—Subject, a dark brown gelding seven years old; autopsy five hours after death; body well nourished; visible mucous membranes somewhat hyperæmic; no enlargement or reddening of the subcutaneous lymph glands; muscles of normal red color; mesenteric lymph glands dark red in color; liver somewhat fatty. Kidneys: the capsule easily detached; surface smooth; pale; glomeruli prominent; pelvis filled with muco-pus or mucous; medullary portion dark red in color; the cortex greyish-yellow; bladder filled with urine which is coffee-colored, with a thick yellowish sediment. The wall of the bladder is covered with striated and punctiform hemorrhages, with, in a few small places, a roughened appearance from loss of mucous membrane. Thoracic organs normal; throat and mouth organs normal; brain and spinal cord normal.

*Microscopic Examination.*—Frozen sections of kidney show the epithelium in the tubes very much swollen and granular with considerable deposition of fat. The glomeruli are greatly distended and the capsule, in places, apparently thickened. There is no thickening of the interlobular connective tissue. Urine contains a small quantity of albumen; also red blood corpuscles and many leucocytes, blood casts, granular casts and urethral, renal and bladder epithelium. Sp. Gr. of urine, 1035.

Autopsy, Case No. 2.—Animals which would evidently have died soon, was killed by bleeding for the purpose of post mortem examination. The urine from this animal was examined before death and found to contain neither albumen nor casts, but many leucocytes. The post mortem made immediately after death revealed no lesions. Brain, spinal cord and sciatic nerve examined. No effusion in any of the cavities.

Microscopic examination of brain, cord and sciatic nerve negative. The urine from some of the other cases was examined but failed to detect albumen or casts.

There have been many cases about here similar to these.

The autopsies which I have made revealed no lesions, save in many cases, swelling and hemorrhage of the mesenteric lymph glands and striated and punctiform hemorrhages under the epicardium, pleura and peritoneum, especially in the pelvic cavity.

Bacteriological investigation made in several of the cases, has been negative.

Dickerhoff in his "Specielle Pathologie und Therapie fur Thierarzte, Vol. 1, F. 498, describes a disease under the heading "Vergiftung durch Kornrade," *Agrostemma Githago*, which resembles closely the disease here described. We failed to find the existence of the corn cockle in the feed at stable where Dr. Dougherty's cases were kept, but at a farm on the eastern shore of Maryland, where they lost several horses from what was to my mind the same disease, I found plenty of cockle in the rye which was used for bedding.

BALTIMORE.

